# Relevance of human metapneumovirus in exacerbations of COPD

**DOI:** 10.1186/1465-9921-6-150

**Published:** 2005-12-21

**Authors:** G Rohde, I Borg, U Arinir, J Kronsbein, R Rausse, TT Bauer, A Bufe, G Schultze-Werninghaus

**Affiliations:** 1 Clinical Research Group "Significance of viral infections in chronic respiratory diseases of children and adults", Department of Internal Medicine III, Pneumology, Allergology and Sleep Medicine, University Hospital Bergmannsheil, D-44789 Bochum, Germany; 2 Clinical Research Group "Significance of viral infections in chronic respiratory diseases of children and adults", Department of Experimental Pneumology, Ruhr-University-Bochum, Bochum, Germany

## Abstract

**Background and methods:**

Human metapneumovirus (hMPV) is a recently discovered respiratory virus associated with bronchiolitis, pneumonia, croup and exacerbations of asthma. Since respiratory viruses are frequently detected in patients with acute exacerbations of COPD (AE-COPD) it was our aim to investigate the frequency of hMPV detection in a prospective cohort of hospitalized patients with AE-COPD compared to patients with stable COPD and to smokers without by means of quantitative real-time RT-PCR.

**Results:**

We analysed nasal lavage and induced sputum of 130 patients with AE-COPD, 65 patients with stable COPD and 34 smokers without COPD. HMPV was detected in 3/130 (2.3%) AE-COPD patients with a mean of 6.5 × 10^5 ^viral copies/ml in nasal lavage and 1.88 × 10^5 ^viral copies/ml in induced sputum. It was not found in patients with stable COPD or smokers without COPD.

**Conclusion:**

HMPV is only found in a very small number of patients with AE-COPD. However it should be considered as a further possible viral trigger of AE-COPD because asymptomatic carriage is unlikely.

## Background

Respiratory viruses play an important role in exacerbations of COPD and this has been increasingly recognised since the application of molecular detection methods [1,2]. The most prevalent viruses detected by polymerase chain reaction so far were respiratory syncytial virus (RSV), Influenza A, Rhinovirus and Parainfluenza 3. Human metapneumovirus (hMPV) is a recently discovered respiratory virus first isolated from a dutch child with lower respiratory tract infection (LRTI)[3]. World wide distribution is probable since it has been isolated in North America[4], Brasil[5], Japan[6], Australia[7] and the United Kingdom[8] from children with LRTI in the winter/spring seasons. Recent publications show a detection rate of 3.9 to 7% in children hospitalized for LRTI [9-12]. When outpatients are investigated for the presence of hMPV the detection rates are higher ranging from 6.2[13] to 12%[14].

HMPV has been recognized as a member of the Paramyxoviridae like RSV and it is not only associated with bronchiolitis in most cases, but also with pneumonia, croup and exacerbations of asthma[14,15], diseases which share some features with COPD. Up to date reports about hMPV in adults are scarce. In a general Canadian population 14.8% of patients of all age groups with acute respiratory tract infections were hMPV positive. Thirty-three percent of hMPV-infected patients were hospitalized and the hospitalization rates were significantly higher among patients below 5 years and those over 50 years of age[16]. In another prospective cohort of adults, hMPV was detected in 4.5% of all illnesses but also in 4.1% of asymptomatic subjects. HMPV was most prevalent in young adults with children and in frail elderly[17]. HMPV infection can be severe since the virus was isolated from the lungs from a previously healthy man who died from acute pneumonia[18]. The role of hMPV in acute exacerbations of COPD (AE-COPD) has been studied recently in outpatients and only low frequencies have been observed[17,19]. Up to now the prevalence of hMPV in patients hospitalized with AE-COPD is unknown. Our aim was therefore to investigate the frequency of detection of hMPV in a prospective cohort of hospitalized patients with AE-COPD and to compare these results to patients with stable COPD and to smokers without COPD.

## Subjects, material and methods

### Subjects

Three different groups were studied. The first group consisted of hospitalized patients with an acute exacerbation of COPD (AE-COPD), the second group were subjects with stable COPD and the third group was composed of smokers without COPD. The groups were defined as previously published[20]. Briefly *AE-COPD patients *suffered from COPD as defined by GOLD[21]. Acute exacerbation was characterized by worsening in dyspnea, cough, and expectoration. A routine posterior-anterior chest radiograph was evaluated on admission by expert radiologists to exclude other other reasons for increased symptoms as pneumonia, tuberculosis, pulmonary fibrosis, bronchiectasis, bronchial carcinoma or congestive heart failure.*Stable COPD patients *did not have an exacerbation within the last 30 days prior to hospital admission and had no changes in therapy within the last 14 days (including inhaled and oral medication) and had been admitted for other medical reasons into departments of internal medicine other than pulmonary care. COPD subjects were recruited in a 2:1 ratio each month in order to prevent seasonal selection bias. *Smokers *have been smoking more than 10 pack-years, could have chronic symptoms like cough and phlegm but did not report dyspnea and did not have bronchial obstruction (FEV_1_/FVC>70%, FEV_1_>80% predicted). None of the smokers had a history of COPD or asthma, nor was using systemic or topic pulmonary medication. The smokers were recruited either from our smoking cessation initiative or by newspaper advertisement.

The study was approved by the ethical committee of the Ruhr-University of Bochum, Germany. Written informed consent was obtained from all patients and control subjects before inclusion in the study.

### Diagnostic methods

Clinical evaluation, spirometric tests, nasal lavage, induced sputum, specimen processing and viral ribonucleic acid (RNA) extraction were carried out as described by Rohde et al[2]. Elution volume was 100 μl. cDNA was generated with random-hexamer primers as previously published[2].

### Detection of hMPV by real-time Reverse Transcriptase PCR

A hMPV-specific real-time RT-PCR designed and evaluated by Maertzdorf et al was used[22]. Primers and probe are localized within the nucleoprotein gene (NL-N) and the presence of a degenerate base within the probe allows detection of all four genetic lineages of hMPV.

The assays were performed using the TaqMan^® ^PCR Core Kit. The final volume was 25 μl containing 500 nM of the forward primer (NL-N-forward (5'-CATATAAGCATGCTATATTAAAAGAGTCTC-3')), 250 nM of the reverse primer (NL-N-reverse (5'-CCTATTTCTGCAGCATATTTGTAATCAG-3')) and 500 nM of the probe (NL-N-probe (5'-FAM-TGYAATGATGAGGGTGTCACTGCGGTTG-TAMRA-3', in which Y is either a C or a T residue). Nuclease-free water was used as negative control and a plasmid containing the N gene of hMPV (kindly provided by James Simon, VIRONOVATIVE, EUR Holding, Erasmus University Rotterdam) was used as a positive control in all PCR runs. Cycling parameters were as follows: 5 min at 95°C, 45 cycles of 30 s at 95°C and 1 min at 60°C. Amplification and detection of RNA from virus isolates or clinical specimens were performed using the GeneAmp^® ^5700 Sequence Detection System (Applied Biosystems). The real-time PCR product was cloned with the QIAGEN^® ^PCR cloning kit (QIAGEN, Hilden, Germany) and this standard plasmid DNA was used for absolute quantification of hMPV viral load. Calculations were performed as previously described for absolute quantification of RSV viral load[23].

### Statistical analysis

The primary objective of this study was to compare the frequency of hMPV detection in respiratory specimens between COPD patients with or without an acute exacerbation and smokers without COPD.

Continuous data were checked for normal distribution using the Kolmogorov-Smirnov test. The data were of non-parametrical distribution and results were expressed as median and range. Differences between groups were assessed by Kruskal-Wallis test. To further analyse significant differences between two individual groups a pair wise comparison by two-sided Mann-Whitney U-test was performed. All significance levels were set to 5%. Data were analysed and processed using SPSS Version 12.0 on a Windows XP operating system.

## Results

### Clinical symptoms

A total of 229 subjects were investigated between October 1999 and June 2004: 130 patients with AE-COPD, 65 patients with stable COPD and 34 smokers without COPD. The clinical characteristics and lung function measurements are summarized in table 1. FEV_1_, FEV_1 _in % of predicted value and FEV_1_/FVC were normal in smokers, significantly decreased in stable COPD patients (all) and further significantly decreased in AE-COPD patients (all p < 0.05 compared to stable COPD and all p < 0.001 compared to smokers).

**Table 1:** Clinical characteristics

	AE-COPD	Stable COPD	Smokers
Age in years	66 [41–80]	66 [45–81]	51 [44–68]
Pack years	32 [2–120]	33 [2–120]	40 [15–92]
FEV_1 _in l	1.0 [0.4–2.2]	1.3 [0.8–2.8]	3.2 [2.0–5.0]
FEV_1 _in %predicted	35.2 [18.7–74.1]	44.2 [22.0–93.7]	100.3 [75.6–141.0]
FEV_1_/FVC in %	46.0 [24.0–69.0]	52.2 [27.0–70.0]	79.8 [72.3–89.6]

AE = Acute exacerbation of COPD

### Prevalence of hMPV

HMPV could be detected in three subjects. All these subjects were AE-COPD patients. The prevalence of hMPV in AE-COPD patients was 2.3%. The virus was simultaneously detected in nasal lavage and induced sputum in one patient only. The viral load was about 100 times higher in nasal lavage than in induced sputum in this patient. Overall the viral load in nasal lavage was about 3.5 times higher compared to induced sputum (for details see table 2). The hMPV positive patients did not differ significantly from other AE-COPD patients when clinical parameters and lung function were analysed. hMPV was detected in the winter season only.

**Table 2:** Viral loads and month of detection of hMPV

Patient No.	VL Nose [copies/ml]	VL Sputum [copies/ml]	Month of detection
1	1.29 × 10^6^	1.28 × 10^4^	3/2001
2	6.73 × 10^3^	-	3/2001
3	-	3.63 × 10^5^	1/2002
Mean VL: [copies/ml]	6.5 × 10^5^	1.88 × 10^5^	

VL = viral load, Nose refers to results form nasal lavage, Sputum refers to results from induced sputum

## Discussion

The main finding of this controlled study investigating the incidence of hMPV in subjects with COPD and smokers without COPD is that this recently discovered respiratory virus was detectable only during exacerbation of COPD. The frequency of detection was very low but in positive cases the viral load was considerable. There was no detection in patients with stable COPD or smokers without COPD.

Our findings are in agreement with other studies in adults. Recently Vicente et al[19] reported about the incidence of hMPV in 89 COPD patients. Five patients (5.5%) were hMPV positive. Two of these patients had to be transferred to hospital. Although this was not a controlled study and not all details of the study are available due to the fact that the data were published in form of a letter, these results support our findings. The incidence of hMPV in this and in our study is low compared to other respiratory viruses. In a similar previous study we found that Picornaviruses were detectable in 36% of AE-COPD patients, Influenza A in 25% and Respiratory syncytial virus in 22%[2]. There is another prospective cohort study of adults in which hMPV was detected in 4.5% of all illnesses. HMPV was most prevalent in young adults with children and in frail elderly from long term care facilities[17]. Unfortunately this report does not specify how many of the elderly patients suffered from COPD. In our asymptomatic smokers without COPD hMPV could not be detected. A recent study investigating nasal secretions from adults with and without respiratory illnesses found hMPV in 5 of 146 ill patient and in none of 158 control subjects, strongly supporting our data[24]. A further recent study found hMPV in two out of 111 adult patients (1.8%) who presented to the emergency department for AE-COPD during 2 winter/spring seasons in Quebec, Canada, also in support of our findings[25]. In a US American study investigating clinical samples collected between 1991 and 1995, hMPV could not be detected at all in 196 patients indicating important geographical and seasonal differences in hMPV prevalence[26]. Taken together the results presented here are in keeping with other studies in adults and add important information on the prevalence of hMPV in hospitalized AE-COPD.

To our knowledge this is the first study analysing the viral load of hMPV in COPD patients. We found a mean of 6.5 × 10^5 ^viral copies/ml in nasal lavage and 1.88 × 10^5 ^viral copies/ml in induced sputum. These values indicate that hMPV may have been the infectious agent triggering exacerbation in these patients. Viral load cut-off values for infectivity in COPD exacerbations have not been studied in detail yet and need further investigation. However, viral loads between 1120 copies/ml in Cytomegalovirus infection in lung-transplant patients[27] and 5.8log_10 _copies/ml in SARS[28] have been considered to indicate infectious disease. Moreover hMPV was only found in acute exacerbation and not in stable disease or in smokers without COPD supporting a triggering role in AE-COPD.

HMPV infection can be severe since it was isolated from the lungs from a previously healthy man who died from acute pneumonia[18]. Our hMPV positive patients did not differ in their clinical characteristics or lung function from the other AE-COPD patients which does not indicate a more severe course of AE-COPD in these patients.

## Conclusion

Taken together this is the first controlled study on the relevance of hMPV in hospitalized AE-COPD. HMPV was detected in a very low frequency but with noticeable viral load in AE-COPD patients. Given that asymptomatic carriage of hMPV is very unlikely it should be considered as another possible trigger of AE-COPD. Since every AE-COPD has considerable impact on the course of the disease and regional outbreaks of hMPV are possible it should be included into future diagnostic and therapeutic considerations.

## List of abbreviations

AE-COPD = Acute Exacerbations of Chronic Obstructive Pulmonary Disease

FEV_1 _= Forced Expiratory Volume in one second

FVC = Functional Vital Capacity

GOLD = Global initiative for chronic Obstructive Lung Disease

LRTI = Lower Respiratory Tract Infection

hMPV = human Metapneumovirus

NL-N = Nucleoprotein gene of hMPV

RSV = Respiratory Syncytial Virus

RT-PCR = Reverse Transcription Polymerase Chain Reaction

RNA = Ribonucleic Acid

## Competing interests

The author(s) declare that they have no competing interests.

## Authors' contributions

GR contributed to the conception and design of the study, analysis and interpretation of data, performed the statistical analysis and drafted the manuscript. IB was responsible for the detection of hMPV by real-time Reverse Transcriptase PCR, analysis of data and helped to draft the manuscript, UA and JK were responsible for recruitment, clinical examination and obtaining the specimens of the patients and control subjects, RR was also responsible for the recruitment of subjects and was involved in the detection of hMPV, TTB participated in the design and coordination of the study, AB contributed to the design of the study, the analysis and interpretation of data and revised the manuscript critically for important intellectual content, GSW has made contributions to conception and design of the study, the interpretation of data and revised the manuscript critically for important intellectual content. All authors read and approved the final manuscript.
